# Effects of long-term cryopreservation on sperm quality and oxidative stress in Manchega rams (*Ovis aries*)

**DOI:** 10.1530/REP-24-0402

**Published:** 2025-09-15

**Authors:** Pedro Javier Soria-Meneses, Alejandro Jurado-Campos, Alejandro Rubio de Juan, Vidal Montoro Angulo, Ana Josefa Soler, José Julián Garde, Manuel Ramón Fernández, María del Rocío Fernández-Santos

**Affiliations:** ^1^ SaBio IREC (CSIC-UCLM-JCCM), Albacete, Spain; ^2^ Instituto Regional de Investigación y Desarrollo Agroalimentario y Forestal de Castilla-La Mancha (IRIAF), CERSYRA, Valdepeñas, Spain; ^3^ INIA-CSIC. Crta de la Coruña, Madrid, Spain; ^4^ Faculty of Pharmacy, UCLM, Albacete, Spain

**Keywords:** genetic resource banks, oxidative stress, cryopreservation, sperm, lipid peroxidation

## Abstract

**In brief:**

This study evaluated the impact of long-term cryopreservation on oxidative stress and sperm quality in Manchega rams, suggesting that sperm quality decline may be due primarily to cryopreservation duration rather than genetic effects.

**Abstract:**

Cryopreservation plays an essential role in artificial insemination protocols and in the establishment of germplasm banks. However, cryopreservation is a key factor contributing to the increase in oxidative stress, potentially leading to deterioration of sperm quality over time. This study examined whether oxidative stress levels differed in Manchega sheep sperm samples stored for up to 25 years. It was hypothesised that these differences could be due to storage time or genetic selection focused on productive traits, given the antagonism between productive and reproductive traits. Frozen sperm samples from 145 rams, all cryopreserved at the same age and processed under identical conditions to minimise variation, were analysed. Sperm viability, intracellular reactive oxygen species production, lipid peroxidation, and chromatin integrity were assessed. The impact of cryopreservation storage duration on sperm quality was evaluated, and genetic trends were estimated using pedigree and genomic data to explore potential associations with reproductive genes. Our results revealed minimal changes in sperm quality parameters across generations. A negative trend was observed in oxidative stress indicators, while sperm viability exhibited a slight positive trend, suggesting that oxidative stress may have a greater impact on older frozen samples. Genetic trend analysis revealed no significant differences, and no associations with reproductive genes were found, which indicates that the deterioration in sperm quality was primarily due to storage time rather than genetic selection. However, sperm quality is influenced by multiple genes not examined in this study, and a genetic effect cannot be completely discarded.

## Introduction

Fertility is not a fixed value; it varies throughout the life of the individual due to factors such as age, physical condition, or environmental stress, among others (Guerrero-Bosagna *et al.* 2013). We now understand that the primary cause of impaired sperm function is multifactorial, involving genetic and environmental factors that often act in conjunction (Aitken *et al.* 2014). Thus, the combination of these factors results in a state of oxidative stress that compromises the functional and structural integrity of these highly specialised cells. This oxidative stress stands out as a major contributor to sperm dysfunction, impacting not only DNA integrity but also limiting the fertilisation potential of these cells due to collateral damage to proteins and lipids in sperm plasma membranes (Aitken *et al.* 2014).

Mammalian spermatozoa are especially susceptible to peroxidative damage due to their high content of polyunsaturated fatty acids (PUFAs), which are highly susceptible to attack by free radicals (Peris *et al.* 2007). Consequently, hydrogen extraction is promoted, initiating lipid peroxidation by generating additional lipid radicals that propagate the peroxidation chain reaction (Christova *et al.* 2004). This process, in turn, produces lysophospholipids that destabilise the sperm plasma membrane, affecting its microarchitecture and altering the functions of integral membrane proteins crucial for sustaining sperm motility, such as ATP-dependent ion pumps and voltage-regulated ion channels (Lundbaek & Andersen 1994). The disruptive impact of peroxidation damage on lipid membrane structure also impairs the ability of spermatozoa to engage in membrane fusion events associated with fertilisation (Aitken *et al.* 1993). Lipid peroxidation chain reactions initiated within spermatozoa can also lead to the generation of a cascade of potent electrophilic aldehyde by-products that bind to various sperm proteins, subsequently affecting sperm function (Ortega-Ferrusola *et al.* 2019). Oxidative stress associated with aldehyde by-products compels spermatozoa to enter the intrinsic apoptotic cascade, starting with a loss of mitochondrial membrane potential and culminating in DNA oxidation, DNA strand breaks, and cell death (De Iuliis *et al.* 2009, Noblanc *et al.* 2013).

Long-term preservation of spermatozoa is a powerful tool for the breeding of agriculturally valuable animals, the conservation of endangered species, and overcoming male infertility (Ramon *et al.* 2013, Caamaño *et al.* 2021). However, due to the unique characteristics of spermatozoa, cryopreservation is a complex technique that requires a delicate balance of multiple factors to achieve optimal results. In the case of ram spermatozoa, the process is even more intricate due to the high proportion of PUFAs present in their membranes, rendering them more vulnerable to cold shock and susceptible to free radical attacks (Salamon & Maxwell 2000). These alterations in the plasma membrane not only impact sperm motility and viability but also affect capacitation and the acrosomal membrane, both crucial for oocyte penetration, ultimately reducing fertility (Watson 2000). Sperm cryopreservation plays a pivotal role in the establishment of genetic resource banks (GRBs). These GRBs are dedicated to indefinitely preserving the genetic material of endangered, rare, valuable, or genetically significant specimens (Leroy *et al.* 2019, Holt 2023). A noteworthy example is the germplasm bank of the Manchega sheep (*Ovis aries*) breed, housing seminal samples collected from 1991 to the present day. In addition, GRBs are indispensable in breeding programs, as they serve to counteract the genetic consequences of incorrect selection practices (Holt & Pickard 1999).

The main objective of animal breeding programs is to enhance the productive capacity of each species and breed. However, the advancements achieved in terms of productivity have been associated with a deterioration in physiological and functional aspects (Veerkamp *et al.* 2002). The white variety of the native Spanish Manchega sheep breed is crucial for the socio-economic sectors of the Castilla-La Mancha region, particularly in dairy production (Calvo *et al.* 2006). Breeding programs for this breed focus on enhancing milk production and quality, including fat and protein content. However, a significant challenge is the recent decline in fertility, with a 12% reduction in artificial insemination success over the past decade. We aim to assess the potential impact of oxidative stress on the germplasm bank in the decline of sperm quality. To this end, we will use specific sperm analysis techniques to evaluate the impact of oxidative stress by means of flow cytometry on sperm samples, which were not routinely used 30 years ago, and which will give us valuable information on the quality of frozen samples (Pena *et al.* 2018, da Silva *et al.* 2023). In addition, flow cytometry allows rapid evaluation of thousands of spermatozoa in a few seconds, resulting in a representative evaluation of the sample. This modern approach replaces other time-consuming and error-prone techniques, such as fluorescence microscopy, which only allows the evaluation of a few hundred spermatozoa and does not provide a representative sample of the ejaculate (Pena *et al.* 2018). Moreover, the use of mixed model computation programs, designed specifically for animal breeding purposes, such as BLUPF90 (Misztal *et al.* 2002), facilitates the estimation of variance components related to reproductive traits of interest (Pelayo *et al.* 2019). In addition, current genetic tools, such as medium- and high-density single nucleotide polymorphism (SNP) chips (genotyping chips), have provided valuable data for mapping domestic species and investigating mutations responsible for genetic variations in economically important traits through genome-wide association studies (GWAS) (Weller *et al.* 2017).

Hence, the aim of this study, which, to our knowledge, is the first to be carried out with such a thorough and exhaustive approach, was to evaluate changes in oxidative stress parameters in successive generations of cryopreserved semen samples, which may be caused by the storage duration after cryopreservation. For that, cryopreserved semen samples from rams that have been selected for milk production aptitude over two decades were used.

We evaluated whether storage time under cryopreservation affects sperm parameters related to oxidative stress, such as free radical production, lipid peroxidation, and their impact on viability and DNA integrity. In addition, the genetic basis of these oxidative stress sperm parameters was evaluated and whether changes in these parameters could be related to genes associated with oxidative stress traits.

## Materials and methods

### Reagents and media

All the chemicals were purchased from Merck (Spain). Biladyl^®^, the freezer extender, was acquired from Minitube (Germany). The fluorescence probes YO-PRO™-1 iodide (YO-PRO-1), propidium iodide (PI), Hoechst 33342 (Hoechst), 5-(and 6)-chloromethyl-2′,7′-dichlorodihydrofluorescein diacetate acetyl ester (CM-H_2_DCFDA), and C11-BODIPY 581/591 were purchased from ThremoFisher Scientific (Spain), with the exception of acridine orange (AO) (Polysciences, Inc., USA). Flow cytometry equipment, software, and consumables were acquired from Beckman Coulter (USA).

The stock solutions of the fluorescence probes were: Hoechst, 16.2 mM in Milli-Q water; PI, 1.5 mM in Milli-Q water; YO-PRO-1, 100 μM in dimethyl sulfoxide (DMSO); CM-H_2_DCFDA, 2 mM in DMSO; and C11-BODIPY 581/591, 0.2 mM in DMSO. The bovine gamete medium (BGM) was composed of 87 mM NaCl, 3.1 mM KCl, 2 mM CaCl_2_, 0.4 mM MgCl_2_, 0.3 mM NaH_2_PO_4_, 40 mM HEPES, 21.6 mM sodium lactate, 1 mM sodium pyruvate, 10 μg/mL phenol red, and 6 mg/mL BSA (pH 7.5). TNE buffer was composed of 0.01 M Tris–HCl, 0.15 M NaCl, and 1 mM ethylenediamine-tetraacetic acid disodium salt dihydrate (EDTA) (pH 7.4); acid-detergent solution for SCSA^®^ 0.17% Triton X-100, 0.15 M NaCl, 0.08 N HCL (pH 1.4); and AO solution 0.1 M citric acid, 0.2 M Na_2_HPO_4_, 1 mM EDTA, 0.15 M NaCl, 6 μg/mL AO, pH 6.0. The freezer extender, Biladyl^®^, was prepared according to the specifications of the manufacturer.

### Sperm collection and experimental design

For this study, semen samples from 145 adult males of the Manchega dairy sheep breed stored in the germplasm bank of the Manchega sheep breed were used. For 25 years, semen samples from males housed at the Regional Centre for Animal Selection and Reproduction (CERSYRA, Spain), which is part of the structure of the Manchega sheep breeding program, were collected using an artificial vagina and frozen following a standard protocol (see below) for storage in the germplasm bank of the breed.

The selection of the 145 rams included in this study was carried out to ensure representation of various generations within the breeding program, facilitating the evaluation of whether changes in sperm parameters occurred during program development. Generations were determined using pedigree information with the ENDOG program (Gutiérrez & Goyache 2005). For the selection of the 145 rams, generation was considered together with year of birth (YOB), so a correlation between both was required, i.e. animals born longer ago belonged to older generations, and those born more recently belonged to more recent generations. In addition, to increase representativeness of the population, for the selection of the rams, a relatedness coefficient below 1/16 between rams was required. For these rams, the average inbreeding coefficient using pedigree information was 0.008. The distribution of the 145 rams across generations was as follows: generation 1 (*n* = 4); generation 2 (*n* = 11); generation 3 (*n* = 22); generation 4 (*n* = 30); generation 5 (*n* = 38); generation 6 (*n* = 26); and generation 7 (*n* = 14). The first and seventh generations were the oldest and most recent, respectively.

All rams housed at CERSYRA, once they reached the number of artificial inseminations to be tested (200–250), underwent semen collection to be frozen and cryopreserved at the germplasm bank. This occurred at the age of 3–3.5 years for all the rams in this study. All rams were managed in the same manner, in the same facilities with the same feed regimen, and were in good health at the time of semen collection. In addition, for organisational reasons, semen freezing was performed during the months of September-October. Before cryopreservation, all semen samples were subjected to the quality control routinely used in the production of semen doses – a semen volume above 0.5 mL; a sperm concentration greater than 3,000·10^6^ spermatozoa/mL; mass motility (0–5 scale) above 4; percentage of motile spermatozoa above 80%; and a sperm quality movement score (0–5) above 4 – and only those ejaculates above the quality thresholds were used.

The cryopreservation protocol used for the Manchega sheep breed was as follows. The samples were cryopreserved using Biladyl^®^, a commercial freezing extender containing 20% egg yolk and 7% glycerol. In brief, the semen was diluted to a concentration of 400 × 10^6^ sperm/mL using Fraction A of Biladyl^®^ and gradually cooled from 30 to 5°C over a 2 h period. Subsequently, the semen samples were further diluted to a concentration of 200 × 10^6^ sperm/mL using Fraction B of Biladyl^®^. After 2 h of equilibration at 5°C, the semen was automatically loaded into 0.25 mL plastic straws and frozen in a programmable biofreezer (IceCube14S-Ver. 1.30; SY-Laboratory Geräte GmbH, Minitüb^®^, Germany) following a specific freezing curve (−20°C/min from 5°C to −100°C and −10°C/min from −100°C to −140°C). The cryopreserved semen was then submerged in liquid nitrogen (LN2) and stored in an ARPEGE 170 LN2 container (Air Liquide Healthcare, Spain), with a capacity of 174 L. This container is located at the breed’s AI centre and is never transported to another location. LN2 levels were checked weekly and refilled twice a month, or sooner if necessary. Finally, after 6 months of cryopreservation, all cryopreserved semen samples (two straws per sample/ejaculate) were re-evaluated to ensure that the freezing process had been correct (unfortunately, we do not have quality data at thawing, as they are only used as a check control, and samples are discarded or kept in the germplasm bank according to the quality observed at this time).

For this study, one straw from each ram was thawed at 37°C for 30 s. Sperm cells viability and apoptosis-like changes, intracellular reactive oxygen species (ROS) production, and sperm lipid peroxidation levels were immediately assessed after thawing (0 h) and after 2 h of incubation at 37°C (2 h). Another straw was used to obtain DNA for genotyping using the ThermoFisher Ovine 50K SNP chip (*Xenetica Fontao* laboratory, Spain), and following the instructions of the manufacturer.

### Fluorescence probes for sperm physiology analysis

#### Sperm viability and apoptotic-like changes assessment

Sperm viability and apoptotic-like changes were evaluated using a combination of 0.04 μM YO-PRO-1 and 3 μM PI in BGM, adding spermatozoa up to 10^6^/mL. YO-PRO-1 is a probe capable of staining cells when there is an increase in membrane permeability (early apoptotic cells), but this is not necessarily indicative of a highly compromised membrane (Peña *et al.* 2005). However, PI binds to DNA in membrane-compromised sperm cells and allows the identification of viability (Gillan *et al.* 2005). After 20 min of incubation in the darkness, sperm samples were evaluated by flow cytometry. Three subpopulations were obtained: viable (YO-PRO-1^−^/PI^−^), apoptotic-like membrane changes (YO-PRO-1^+^⁄ PI^−^), and dead (YO-PRO-1^+^/PI^+^) sperm cells.

#### Intracellular ROS production assessment

ROS production was measured using the CM-H_2_DCFDA fluorescent probe. This probe crosses the plasma membrane, is held after intracellular esterases cleave the acetate groups, and emits green fluorescence after oxidation (Dominguez-Rebolledo *et al.* 2010*a*). Sperm samples were stained with 0.2 μM CM-H_2_DCFDA in BGM and incubated at 37°C (30 min, darkness). Then, samples were stained with 3 μM PI in order to discriminate viable and dead cell populations. After 10 min of incubation in the darkness, the samples were run through a flow cytometer to a final concentration of 10^6^ spermatozoa/mL. The mean intensity of fluorescence (MIF) of CM-H_2_DCFDA of the viable sperm subpopulation (PI^−^) was noted. One of the limitations of this probe is that it is non-specific (Dominguez-Rebolledo *et al.* 2010*a*), whereas others detect specific oxidant species, e.g. BODIPY probes detecting lipid peroxidation of membranes (Dominguez-Rebolledo *et al.* 2010*b*).

#### Lipid peroxidation assessment

To estimate the susceptibility of sperm to lipid peroxidation, a C11-BODIPY 581/591 (BODIPY) fluorescent probe was used. BODIPY emits orange fluorescence in its non-oxidised state, shifting to green fluorescence when peroxidised (Dominguez-Rebolledo *et al.* 2010*b*). Samples were incubated in the darkness with 0.02 μM BODIPY probe in BGM at 37°C (30 min, darkness). Then, samples were stained with 3 μM PI viability probe. After 10 min of incubation in the darkness, the samples were run through a flow cytometer to a final concentration of 10^6^ spermatozoa/mL. The MIF of BODIPY of the viable sperm subpopulation (PI^−^) was noted.

#### Sperm chromatin integrity assessment

The technique used to assess sperm chromatin status was SCSA^®^. Semen samples were diluted in TNE buffer to a final concentration of 2 × 10^6^ cells/mL, frozen immediately in liquid nitrogen, and stored in a freezer at −80°C until analysis. Samples were thawed in crushed ice. Acid-induced denaturation of DNA *in situ* was achieved by adding 0.4 mL of an acid-detergent solution to 200 μL of the sample. After 30 s, the cells were stained by adding 1.2 mL of an AO solution, and the stained samples were analysed by flow cytometry exactly 3 min after adding the AO solution. A tube with 0.4 mL of detergent-acid solution and 1.2 mL of AO solution was run through the system before any samples and between samples. At the beginning of each session, a standard semen sample was run through the cytometer, and settings were adjusted so that mean fluorescence values (0–1,023 linear scale) for FL-1 (green fluorescence) and FL-3 (red fluorescence) were 475 and 125, respectively. Results of the DNA denaturation test were processed to obtain the DFI, i.e. the ratio of red fluorescence versus total intensity of the fluorescence (red/(red + green) × 100) for each spermatozoon, representing the shift from green to red fluorescence. High values of DFI indicate chromatin abnormalities. Flow cytometry data were processed to obtain %DFI (% of spermatozoa with DFI >25) and %HDS, which is the percentage of spermatozoa with green fluorescence higher than channel 600 of 1,024 channels (Evenson *et al.* 2002).

#### Flow cytometry analysis

**Figure 1:**
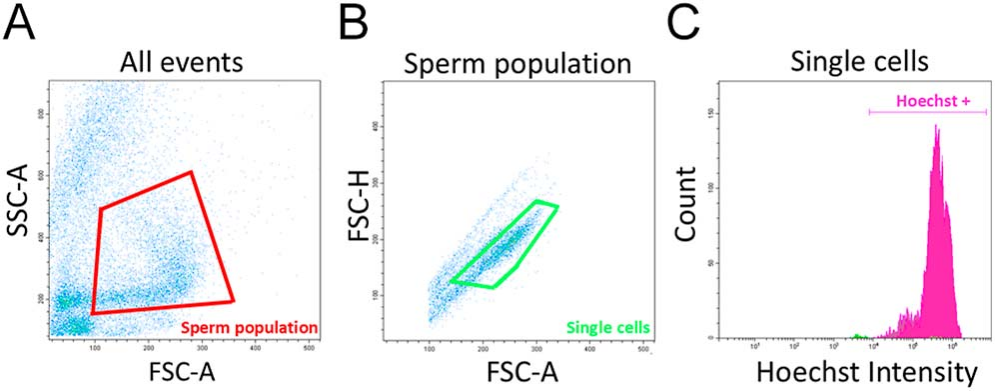
Cytograms and histogram of flow cytometry analyses for assessing spermatozoa physiology. (A) Cytogram of FSC (forward scatter of the laser light) vs SSC (side scatter of the laser light) dot plot, used to rule out non-sperm events. (B) Cytogram of FSC-area (FSC-A) vs FSC-height (FSC-H) dot plot, used to exclude doublets and clumps. (C) Histogram of Hoechst 33342 intensity. Hoechst was employed to identify DNA-containing events; sperm cells were detected by positive Hoechst staining.

All sperm physiology analyses with fluorescent probes were evaluated with a CytoFlex S cytometer (Beckman Coulter, Inc., USA) equipped with a 405 nm laser for excitation of Hoechst and a 488 nm laser for excitation of YO-PRO-1, PI, CM-H_2_DCFDA, and BODIPY. Hoechst fluorescence was read using a 450/45 band-pass filter; YO-PRO-1, CM-H_2_DCFDA, and BODIPY fluorescence were read using a 525/40 band-pass filter; and PI fluorescence was read using a 610/20 band-pass filter. Non-sperm events were discarded by gating in a FSC (forward scatter of the laser light)/SSC (side scatter of the laser light) dot plot, based on differences in size and complexity among spermatozoa and debris. FSC-area (FSC-A) vs FSC-height (FSC-H) dot plot was used to exclude doublets and clumps. Hoechst was employed to identify DNA-containing events; sperm cells were detected by positive Hoechst staining (Fig. 1). Sperm cells were run through the instrument at 150–300 cells/s, collecting data from 10,000 cells. The analysis of the flow cytometry data was carried out using the software CytoExpert version 2.3.0.84 (Beckman Coulter, Inc., USA). This software makes it possible to compare the emissions of the probes collected by the photodetectors in dot plots, making it possible to identify different cell populations according to the fluorescence intensity in each event.

SCSA^®^ was performed on a Cytomics FC-500 (Beckman Coulter, Inc., USA) controlled with the MXP software (v.3) and equipped with a 488 nm Argon-Ion laser for excitation of AO. A 530/28 band-pass filter and FL-1 photodetector were used to detect AO green fluorescence, while a 620/40 filter and FL-3 photodetector were used to detect AO red fluorescence. The data were saved in flow cytometry standards v.2 files. Analysis of the data was carried out using WEASEL v.2.6 (F. Battye, WEHI, Australia).

### Statistical analysis

The R (v 4.3.2) statistical package was used to perform the statistical analysis of this study (Team 2021). To assess the changes that may have occurred over the past two decades in the Manchega sheep breed’s breeding program, we conducted a longitudinal study. We employed regression analysis to understand how variations in the flow cytometry sperm parameters mentioned earlier could be attributed to the generation to which each animal belonged, this being associated with the storage duration but also with possible differences in the genetic background of the rams belonging to different generations as a consequence of the selection decisions taken in the breeding program of the breed. We preferred using generation information over YOB because it allowed for a more suitable grouping of animals. Generation was determined using pedigree information with the ENDOG program. For that, YOB was also considered. In addition, for this study we made sure that there was a correlation between generation and YOB. As the reviewer points out, there is not always a match between generation and YOB. Thus, an animal born in 2015 could belong to the first generation if no information about its parents was available. For this reason, for the choice of the 145 rams used in this study, this correlation was required, so that animals born longer ago belonged to older generations, and those born more recently belonged to more recent generations. It is important to note that for this study, we selected animals in a way that ensured distinct generations, meaning that any animal in a specific generation was born after those in previous generations and before those in later generations, i.e. animals born longer ago belonged to older generations, and those born more recently belonged to more recent generations.

The regression model considered generation as a fixed effect, both as a categorical factor (with one level per generation, from 1 to 7) and as a quadratic polynomial approach. In addition, we accounted for the incubation period (0 and 2 h after thawing) and treated the identification of the ram as a random effect to address variability among and within individuals. Other factors were not included in the model since the freezing protocol used in the breed remained consistent over the years, as did the thawing conditions.

To gain deeper insights into the potential genetic basis of the observed variations in these sperm parameters across generations, we calculated genetic trends for these traits. We utilised pedigree information provided by the national association of selective breeders of Manchega sheep (AGRAMA) and estimated breeding values using the BLUPF90 suite (Misztal *et al.* 2002). These genetic trends were standardised by dividing them by the additive genetic standard deviation, allowing for comparisons among traits. Furthermore, to explore the genetic determinants of these observed trait changes, we investigated whether alterations occurred in genes known to be associated with the evaluated sperm traits. The selection of these genes was based on different studies found in the literature that have reported specific genes related to a number of oxidative stress traits of interest. There are many other genes that could be related to oxidative stress traits or other traits that determine fertilisation outcome, but the idea was to focus on a few of them and see if changes in allele/genotypic frequencies occurred. We obtained genomic data from the breeders’ association, which included SNPs located within these genes, and assessed whether there were significant changes in allele and genotypic frequencies over the generations. The specific genes examined are listed in Table 1.

**Table 1:** Genes identified as related to the sperm traits evaluated. Genes related to sperm parameters such as viability (VIA), intracellular ROS production (ROS), and lipid peroxidation levels (LPO), as well as the chromosome (Chr) where they are located, the gene symbol, and the biological process in which they participate.

Parameter/Chr	Gene symbol	Gene name	Biological process, molecular function, phenotype
VIA			
1	*PDCD1*	Programmed cell death 1	Immune-inhibitory receptor expressed in activated T cells. Regulation of T-cell functions
3	*ATP2B1*	ATPase plasma membrane Ca^2+^ transporting 1	Enable P-type calcium transporter activity. Negative regulation of cytosolic calcium ion concentration
13	*SMOX*	Spermine oxidase	Enables polyamine oxidase activity. Involved in spermine catabolic process
16	*NADK2*	NAD kinase 2, mitochondrial	Enable NAD + kinase activity and protein homodimerisation activity. NAD metabolic process
19	*CMTM6*	CKLF-like MARVEL transmembrane domain containing 6	Endocytic recycling; protein transport; and regulation of protein stability
ROS			
2	*LOC101107420*	Phospholipase A2, membrane associated	Catalyses the release of fatty acids from phospholipids. Phospholipid remodelling, arachidonic acid release, leukotriene and prostaglandin synthesis, Fas-mediated apoptosis, and transmembrane ion flux in glucose-stimulated B-cells
3	*PLB1*	Phospholipase B1, membrane-associated	Encodes a membrane-associated phospholipase that displays lysophospholipase and phospholipase A2 activities through removal of sn-1 and sn-2 fatty acids of glycerophospholipids
7	*PCK2*	Phosphoenolpyruvate carboxykinase 2, mitochondrial	Catalyses the conversion of oxaloacetate to phosphoenolpyruvate in the presence of guanosine triphosphate (GTP)
8	*MTO1*	Mitochondrial tRNA translation optimization 1	Encodes a mitochondrial protein thought to be involved in mitochondrial tRNA modification
13	*BAMBI*	BMP and activin membrane bound inhibitor	Positive regulation of epithelial to mesenchymal transition; of signal transduction; and transforming growth factor beta receptor signalling pathway
15	*CLMP*	CXADR-like membrane protein	Adipocyte maturation and development of obesity
16	*LOC101113624*	ATP synthase subunit f, mitochondrial	Encodes a subunit of mitochondrial ATP synthase. Catalyses ATP synthesis, using an electrochemical gradient of protons across the inner membrane during oxidative phosphorylation
LPO			
1	*DAZL*	Deleted in azoospermia like	Encodes potential RNA binding proteins that are expressed in prenatal and postnatal germ cells of males and females
4	*MAGI2*	Membrane associated guanylate kinase, WW and PDZ domain containing 2	Enables SMAD binding activity and type II activin receptor binding activity
9	*DCSTAMP*	Dendrocyte expressed seven transmembrane proteins	Cellular response to cytokine stimulus; myeloid leucocyte differentiation; and positive regulation of bone resorption
14	*CATSPERG*	Cation channel sperm associated auxiliary subunit gamma	Subunit of the CATSPER sperm calcium channel, which is required for sperm hyperactivated motility and male
14	*SPATA2L*	Spermatogenesis associated 2 like	Carbohydrate metabolic process; cell differentiation; and spermatogenesis
18	*CATSPER2*	Cation channel sperm associated 2	Cation channel proteins that localize to the flagellum of spermatozoa. Defects at this locus causes male infertility
19	*ATP2B2*	ATPase plasma membrane Ca^2+^ transporting 2	Enables calcium-dependent ATPase activity. Involved in cochlea development and regulation of cytosolic calcium ion concentration
19	*P4HTM*	Prolyl 4-hydroxylase, transmembrane	Degradation of hypoxia-inducible transcription factors under normoxia. It plays a role in adaptation to hypoxia and may be related to cellular oxygen sensing
25	*GHITM*	Growth hormone inducible transmembrane protein	Inner mitochondrial membrane organization and negative regulation of release of cytochrome c from mitochondria

Chr, chromosome; VIA, viability; ROS, intracellular ROS production; LPO, lipid peroxidation.

## Results

### Sperm viability analysis results

**Figure 2:**
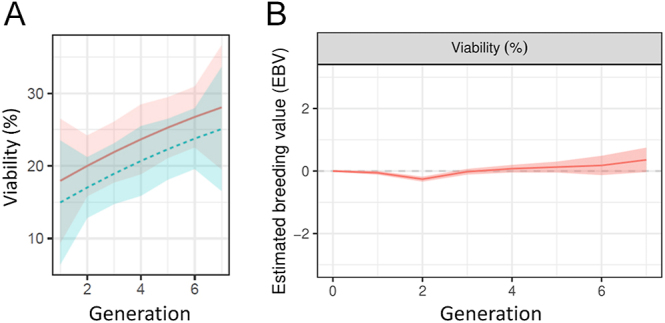
Trends of sperm viability over generations. (A) Trend of the percentage of sperm cell viability (YO-PRO-1^−^/PI^−^, %). The solid red line represents the trend curve of the results after 0 h of incubation of the samples, while the dotted blue line represents the trend of the results after 2 h of incubation of the samples at 37°C. (B) Estimated genetic trends (standardised values) for sperm cell viability (YO-PRO-1^−^/PI^−^, %) over generations.

**Figure 3:**
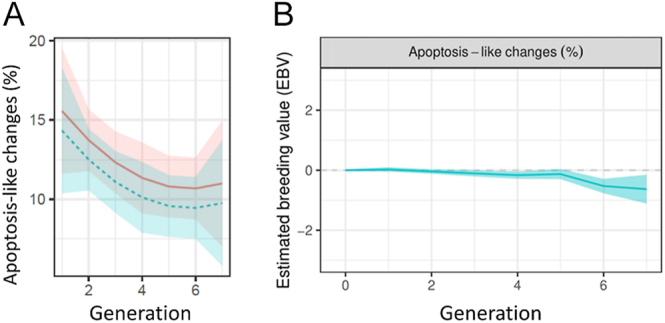
Trends of sperm apoptotic-like changes over generations. (A) Trends of the percentage of sperm apoptotic-like changes (YO-PRO-1^+^/PI^−^, %). The solid red line represents the trend curve of the results after 0 h of incubation of the samples, while the dotted blue line represents the trend of the results after 2 h of incubation of the samples at 37°C. (B) Estimated genetic trends (standardised values) for sperm apoptosis-like changes (YO-PRO-1^+^/PI^−^, %).

The percentage of viable spermatozoa exhibited a consistent linear increase across generations, with a linear trend of 2.27% per generation (Fig. 2A). Conversely, the percentage of spermatozoa displaying apoptosis-like changes showed a decreasing linear trend of −2.47% per generation (Fig. 3A). A significant reduction (*P* < 0.05) in the percentages of viable and apoptosis-like changes was noted after a 2 h incubation period. Motility results are shown in Table 2. When assessing estimated genetic trends for these two traits (Figs 2B and 3B), there were no significant changes in the percentage of viable spermatozoa, while a slight decrease in genetic values was observed for the more recent generations (5–7) in the case of apoptosis-like changes. Our analysis sought to identify any significant shifts in allele and genotypic frequencies for genes related to viability, such as the PDCD1 gene (programmed cell death 1) involved in the regulation of T-cell functions, ATP2B1 gene (ATPase plasma membrane Ca^2+^ transporting 1) involved in the negative regulation of cytosolic calcium ion concentration, and SMOX gene (spermine oxidase) involved in spermine catabolic process. No significant alterations in the allele and genotypic frequencies of the markers associated with genes related to sperm viability and apoptosis-like changes were observed.

**Table 2:** Results of sperm viability, intracellular ROS production, and lipid peroxidation in viable sperm cells by flow cytometry with regard to the ram generations studied, immediately after thawing (hour 0) and after two hours incubation at 37°C (hour 2). The results are presented as mean ± SEM.

	Generation						
	1	2	3	4	5	6	7
Viable (%)							
0 h	21.88 ± 4.28	19.93 ± 13.43	21.21 ± 8.77	23.54 ± 11.91	25.12 ± 11.29	27.88 ± 13.24	26.79 ± 9.99
2 h	20.45 ± 8.90	14.69 ± 10.13	17.12 ± 7.99	20.64 ± 11.53	23.57 ± 11.72	24.89 ± 13.70	22.82 ± 8.32
Apoptotic-like change (%)							
0 h	16.49 ± 4.98	13.11 ± 5.14	12.49 ± 6.43	10.77 ± 5.93	11.62 ± 5.80	10.41 ± 4.91	10.48 ± 5.71
2 h	15.23 ± 3.69	12.20 ± 3.45	11.44 ± 5.20	9.47 ± 4.82	9.76 ± 5.23	9.66 ± 5.23	9.75 ± 4.87
ROS production (MIF)							
0 h	1,740.35 ± 197.80	1,587.86 ± 107.34	1,693.12 ± 224.76	1,647.63 ± 237.32	1,582.45 ± 142.79	1,521.08 ± 218.71	1,482.74 ± 138.43
2 h	1,590.53 ± 90.71	1,604.50 ± 112.36	1,708.12 ± 269.37	1,650.10 ± 208.88	1,562.33 ± 200.45	1,617.24 ± 210.57	1,553.31 ± 170.24
LPO (MIF)							
0 h	1,449.33 ± 54.86	1,568.14 ± 122.02	1,575.65 ± 289.52	1,502.97 ± 176.73	1,386.92 ± 153.00	1,332.84 ± 100.78	1,371.54 ± 142.10
2 h	1,398.20 ± 32.05	1,536.93 ± 147.68	1,606.74 ± 350.86	1,520.05 ± 174.18	1,459.00 ± 254.14	1,412.86 ± 154.79	1,951.59 ± 1,881.34
DFI (%)							
0 h	1.81 ± 0.51	1.24 ± 0.55	1.09 ± 0.67	0.92 ± 1.00	0.87 ± 0.56	1.06 ± 0.49	0.98 ± 0.31
2 h	1.89 ± 0.58	1.34 ± 0.83	1.14 ± 0.56	0.98 ± 0.54	0.96 ± 0.54	1.11 ± 0.48	1.21 ± 0.55
HDS (%)							
0 h	11.36 ± 19.93	10.37 ± 1.65	11.24 ± 2.24	12.16 ± 2.02	13.09 ± 2.75	13.71 ± 2.92	12.75 ± 1.97
2 h	11.10 ± 0.90	10.32 ± 3.33	11.53 ± 2.38	13.05 ± 2.05	13.52 ± 2.53	14.33 ± 3.45	13.62 ± 2.91

Viable, YO-PRO^−^/PI^−^. Apoptotic-like change, YO-PRO^+^/PI^−^. ROS production, intracellular ROS production in viable spermatozoa using the mean intensity of fluorescence (MIF) of CM- H_2_DCFDA. LPO, lipid peroxidation in viable spermatozoa using the mean intensity of fluorescence (MIF) of BODIPY. DFI, DNA fragmentation index. HDS, high DNA stainability.

### Intracellular ROS production assessment results

**Figure 4:**
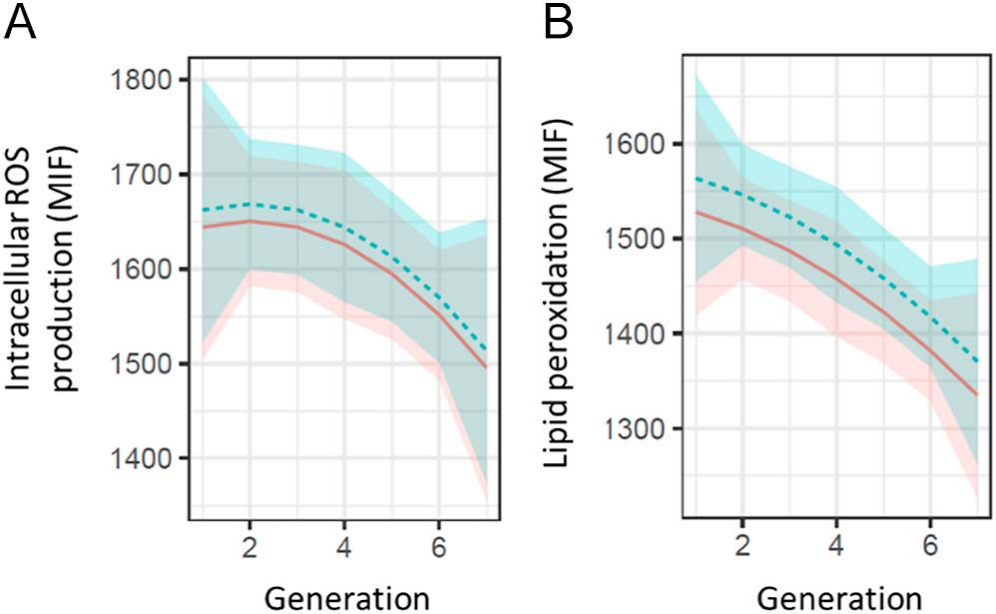
Trend in intracellular ROS production and lipid peroxidation over generations. The solid red line represents the trend of the results after 0 h of incubation of the samples, while the dotted blue line represents the results after 2 h of incubation of the samples at 37°C. (A) Intracellular ROS production (MIF). (B) Lipid peroxidation (MIF).

**Figure 5:**
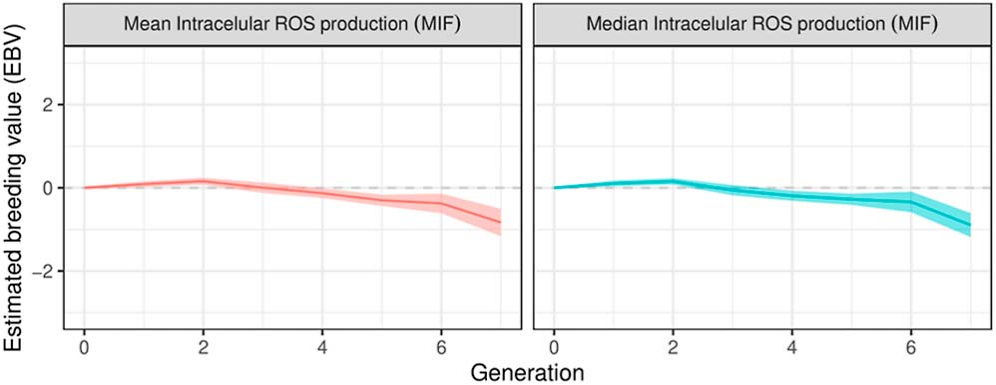
Estimated genetic trends (standardised values) for intracellular ROS production levels over generations. Mean and median values were considered.

Results of intracellular ROS production evaluation showed a significant decreasing linear trend (*P* < 0.05) between the intracellular ROS production and the generation (Fig. 4A), with a decrease of −25.7 MIF per generation, resulting in lower levels of ROS in recent generations. No significant differences (*P* ≥ 0.05) were observed after 2 h of incubation for intracellular ROS production. Intracellular ROS production results are shown in Table 2. The assessment of genetic trends across generations revealed no notable alterations for this trait; only for the last generation was a significant decrease in intracellular ROS production observed (Fig. 5). When examining the allele and genotypic frequencies of the markers related to genes of interest for this trait, such as the *LOC101107420* gene (phospholipase A2, membrane associated) involved in catalysing the release of fatty acids from phospholipids, *PLB1* gene (phospholipase B1, membrane associated) involved in encoding a membrane-associated phospholipase that deploys lysophospholipase and phospholipase A2 activities by removing fatty acids from glycerophospholipids, and *PCK2* gene (phosphoenolpyruvate carboxykinase 2, mitochondrial) involved in catalysing the conversion of oxaloacetate to phosphoenolpyruvate in the presence of guanosine triphosphate (GTP), no significant changes were observed in the allele and genotypic frequencies of the markers related to genes of interest for intracellular ROS production traits (Table 1).

### Lipid peroxidation assessment results

**Figure 6:**
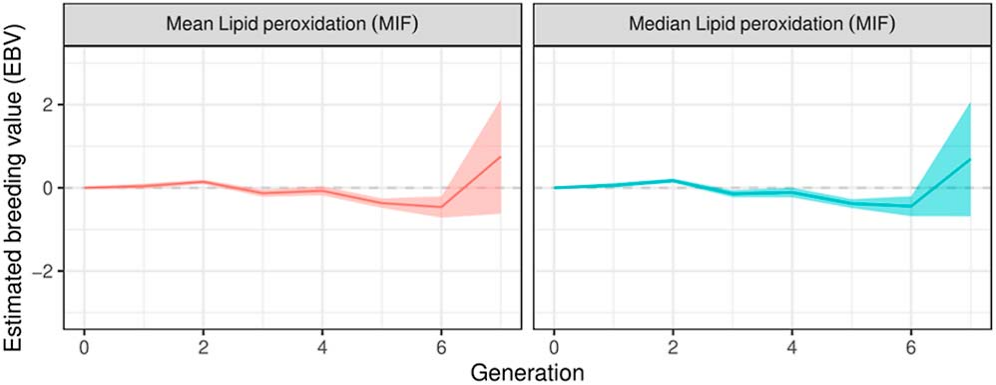
Estimated genetic trends (standardised values) for lipid peroxidation levels over generations. Mean and median values were considered.

Similar results were observed for lipid peroxidation levels, with a decreasing linear trend (*P* < 0.05) of −8.8 MIF per generation (Fig. 4B). Incubation for 2 h resulted in a significant increase in lipid peroxidation levels. Significant differences (*P* < 0.05) were observed after 2 h of incubation for lipid peroxidation levels. No significant changes were observed in the genetic trend for lipid peroxidation levels over generations; for the last generation, a marked increase (not significant) was observed for this trait (Fig. 6). Lipid peroxidation results are shown in Table 2. In addition, no significant changes were observed in the allele and genotypic frequencies of the markers related to genes of interest for sperm levels of lipoperoxidation trait, such as the *CATSPER2 *gene (cation channel sperm associated 2) which encodes cation channel proteins that localise to the flagellum, *ATP2B2* gene (ATPase plasma membrane Ca^2+^ transporting 2) which enables calcium-dependent ATPase activity, and *P4HTM* gene (prolyl 4-hydroxylase, transmembrane) involved in degradation of hypoxia-inducible transcription factors under normoxia (Table 1).

### Sperm chromatin integrity assessment results

**Figure 7:**
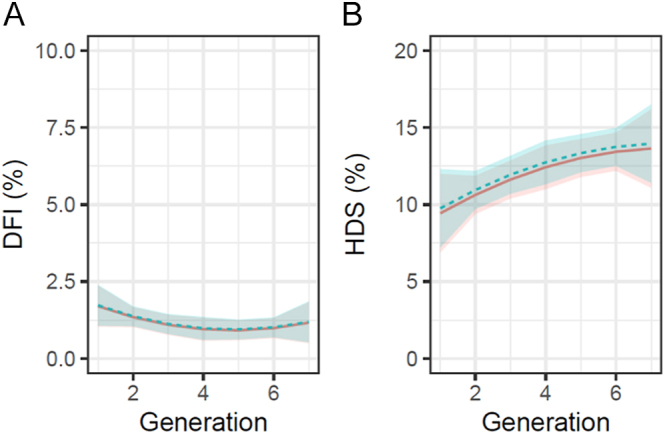
Trend of sperm DNA fragmentation index (%DFI) and high DNA stainability (%HDS) over generations. The solid red line represents the trend curve of the results after 0 h of incubation of the samples, while the dotted blue line represents the trend of the results after 2 h of incubation of the samples at 37°C. (A) DNA fragmentation index (DFI, %). (B) High DNA stainability (HDS, %).

**Figure 8:**
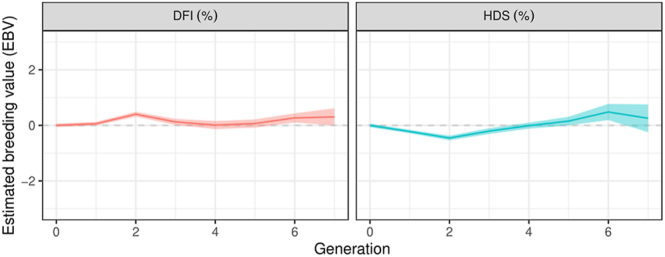
Estimated genetic trends (standardised values) of sperm DNA fragmentation index (%DFI) and high DNA stainability (%HDS) over generations: DNA fragmentation index (DFI, %); high DNA stainability (HDS, %).

In the analysis of SCSA^®^ results, two parameters were considered: %DFI and %HDS. No significant changes were observed for the %DFI over generations (*P* ≥ 0.05). %DFI and %HDS results are shown in Table 2. For the %HDS parameter, a significant linear trend was observed (*P* < 0.05), with an increase of 1.49% per generation (Fig. 7). For the %DFI variable, no changes were observed after incubation for 2 h, but for the %HDS. Estimation of genetic trends over generations showed no significant changes for both traits (Fig. 8). No markers related to genes of interest for DNA integrity traits were observed (Table 1).

## Discussion

The main objective of the present study was to investigate potential variations in oxidative stress levels and their possible impact on the structure and functionality of spermatozoa over the last decades of the Manchega sheep sperm bank. The storage protocol has remained unchanged since the establishment of the germplasm bank in this breed, and so our hypothesis was that changes in sperm quality of frozen semen occurring over the years could be due to: i) phenomena occurring during the storage of cryopreserved samples, one of these phenomena being the negative impact of oxidative stress on spermatozoa, as has been demonstrated in studies carried out in different species (Martin Muñoz *et al.* 2015, Banday *et al.* 2017); and ii) due to a decrease in the ability of sperm to cope with the cryopreservation process as a consequence of a correlated antagonist genetic response in the ram within the Manchega sheep breeding program. To the best of our knowledge, this is the first study to investigate the impact of oxidative stress on germplasm bank samples frozen for over 30 years, conducting a multi-generational study over a total of 25 years.

An increase in ROS levels can lead to deleterious effects on sperm structure and function (Aitken *et al.* 2016). Therefore, in this study, we carried out an analysis of intracellular ROS production, which resulted in a negative linear trend in ROS production with respect to generation. This means that sperm samples from more recent generations had lower levels of intracellular ROS than samples from older generations. Therefore, semen samples from older generations are more susceptible to increased lipid peroxidation and DNA damage, which can lead to irreversible damage to the sperm cell and a loss of the fertilising capacity of the semen sample (Aitken *et al.* 2012, Del Olmo *et al.* 2015).

The analysis of lipid peroxidation aligns with the results observed in intracellular ROS production assessments. These findings reveal a consistent negative trend in lipid peroxidation across generations. PUFAs are pivotal in maintaining membrane fluidity and flexibility, which are essential for membrane fusion events associated with fertilisation (Christova *et al.* 2004, Peris *et al.* 2007). However, the presence of double bonds in PUFA molecules renders them susceptible to free radical attacks, initiating a cascade of lipid peroxidation. Consequently, these attacks ultimately impair sperm function through oxidative stress, accompanied by the generation of cytotoxic byproducts, such as 4-hydroxynonenal (4-HNE) and malondialdehyde (MDA) (Ayala *et al.* 2014).

A positive trend was observed in the percentage of viable spermatozoa relative to generation, while a negative trend emerged in spermatozoa entering early apoptosis relative to generation. This observation could be linked to the lipid peroxidation study results, as increased lipid peroxidation disrupts the phospholipid structure and alters membrane fluidity, leading to cell damage. Subsequently, this damage triggers a cascade of apoptosis, ultimately resulting in sperm cell death (Chelucci *et al.* 2015). In addition, the cryopreservation process, which is essential for the establishment of GBRs, leads to increased oxidative stress and decreased viability, which has been demonstrated in different species and breeds such as pig (Caamaño *et al.* 2021), bull (Vigolo *et al.* 2022), horse (Peña & Gibb 2022), ram (Peris *et al.* 2007), and human (Thomson *et al.* 2009). Despite efforts to optimise of freezing protocols, a reduction of approximately 50% in viability is commonly observed when comparing cryopreserved semen to fresh semen in several animal species (Roca *et al.* 2006), including rams (García *et al.* 2018). These findings are consistent with our study, where viability of sperm cells rarely exceeded 50% in any of the ram generations studied.

Moreover, assessing potential DNA damage is crucial because such damage can lead to decreased success in *in vitro* fertilisation (Aitken *et al.* 2009, Noblanc *et al.* 2013). Since increased oxidative stress conditions can lead to oxidation of bases in DNA (Aitken *et al.* 2013), it could also lead to DNA destabilisation and increased susceptibility of DNA to hydrolysis, resulting in the formation of single-stranded DNA, which can be assessed by SCSA^®^. The results for chromatin fragmentation (%DFI) consistently showed very low values across generations. These results could be explained by the highly compacted chromatin of ram spermatozoa (Perreault *et al.* 1988), which makes it very difficult to detect DNA damage (Soria-Meneses *et al.* 2022, Jurado-Campos *et al.* 2023). However, our results for %HDS showed a positive linear trend with respect to generation, indicating that more recent generations have higher %HDS values than older ones. In contrast to %DFI, the clinical value of the %HDS is being discussed, with some researchers advocating its practical significance (Evenson *et al.* 2020, Vaughan *et al.* 2020), while others question its clinical utility (Mohammadi *et al.* 2020, Lu 2022).

In turn, these sperm samples were obtained from rams that have been selected for increased milk production; therefore, it would be expected that there would be a correlated genetic response causing alterations leading to increased oxidative stress, and consequently a loss of quality of the samples. Differences across generations in intracellular ROS levels and lipid peroxidation were not significant. However, trend analyses of these parameters revealed slight variations between generations. To determine whether these differences were influenced by the breeding program itself, we estimated the observed genetic responses for these traits. In this study, no significant genetic changes were observed for any of the sperm parameters evaluated, although for some of them a change in the genetic trend has been observed in the most recent generations. Thus, sperm levels of intracellular ROS or the percentage of spermatozoa with apoptosis-like changes showed this downward trend. These changes in sperm levels of ROS, if maintained, could result in higher reproductive capacity (Aitken 2020, Pavuluri *et al.* 2024). Regarding allele and genotypic frequencies of markers associated with genes of interest, no significant changes were observed among the generations. Therefore, even though these no significant changes are evident at present, it is interesting to monitor the genetic trend for these parameters and, if it is maintained over time, the causes of this trend should be studied in more detail and, perhaps, breeding strategies to maintain it should be proposed. In addition, it is important to note that only a small set of genes that have been previously reported to be related to sperm fertility and sperm quality have been considered in this study. For these genes, no significant differences have been observed. However, many other genes could play an important role in the observed differences in oxidative stress or sperm quality between individuals of different generations, so we cannot completely rule out the existence of a genetic basis.

The slight differences observed in the quality of samples from different generations have led us to consider that these subtle differences can be attributed to the time during which the samples have been cryopreserved. Regarding the relationship between preservation time and sample quality, it is important to note that various studies on different species have yielded contradictory findings. Some studies indicate no degradation in sample quality over time (Edelstein *et al.* 2008, Fraser *et al.* 2014, Malik *et al.* 2015), while others report a decline in sample quality (Yogev *et al.* 2010, Chrenek *et al.* 2017, Huang *et al.* 2019).

Finally, this study also highlights the importance of new technologies for the analysis of sperm parameters, such as flow cytometry. The utilisation of these cutting-edge techniques for the analysis of frozen sperm samples three decades ago, when these techniques were still unfamiliar and not commonly found in animal reproduction laboratories, now allows us for a much deeper study of germplasm banks, which may unveil new insights into the cryopreservation processes, especially concerning oxidative stress processes.

In conclusion, germplasm sperm banks and breeding programs are not exempt from the issues associated with increased oxidative stress. Cryopreservation plays a significant role in these kinds of programs, but the process often entails a series of effects, including oxidative stress, which can harm sperm cells. Although all our data have shown that the changes over time have been minimal, they have indeed occurred. These results seem to suggest a trend toward lower intracellular ROS production and, consequently, reduced levels of lipid peroxidation, which could be related to higher sperm viability in more recent generations. Based on our results, we cannot conclude that these changes are related to selection decisions made in the breeding program and, therefore, have, in part, a genetic basis. However, as discussed above, we cannot completely rule out a genetic basis, as many other genes not considered in this study play an important role in resistance of sperm to cryopreservation. Therefore, in view of our results, we can conclude that the observed changes are due, in part, to storage time. In order to avoid problems associated with oxidative stress during cryopreservation, it would be advisable to routinely use different pre- or post-freezing antioxidant strategies.

## Declaration of interest

The authors declare that they have no known competing financial interests or personal relationships that could have appeared to influence the work reported in this paper.

## Funding

Financial support for this work was from grants SBPLY/21/180501/000111 and SBPLY/17/180501/000369 funded by the Education and Science Council of Junta de Comunidades de Castilla-La Mancha, and grants GL2017-85603-P, PID2020-120281RB-100, and PID2020-117788RB-100 funded by MICIU/AEI/10.13039/501100011033 (Spanish Ministry of Science, Innovation and Universities) and the European Union. PJS-M was supported by a JCCM scholarship, and AJ-C was supported by a UCLM scholarship.

## Author contribution statement

PJS-M and MdRF-S contributed to conceptualisation. PJS-M, AJ-C, MR-F and MdRF-S helped in methodology. PJS-M, AJ-C, ARdJ and MR-F were responsible for software. PJS-M and MdRF-S were responsible for validation. PJS-M, AJ-C, MR-F and MdRF-S helped in formal analysis. MdRF-S was responsible for resources. PJS-M and AJ-C contributed to data curation. PJS-M was responsible for original draft preparation. PJS-M, VM-A, AJS, MR-F, and MdRF-S helped in writing review and editing. JJG, MR-F, and MdRF-S were responsible for supervision. VM-A, AJS, JJG, MR-F and MdRF-S contributed to funding acquisition.

## Ethics approval

Not applicable. The manipulation of the animals in this study is part of the usual practice in livestock farming. Only frozen sperm samples that form part of the genetic breeding programme of the national association of selective breeders of Manchega sheep (AGRAMA) were used, without any manipulation of the animals.
